# Regression of corneal opacity and neovascularization in Stevens-Johnson syndrome and Toxic Epidermal Necrolysis with the use of prosthetic replacement of the ocular surface ecosystem (PROSE) treatment

**DOI:** 10.1016/j.ajoc.2022.101520

**Published:** 2022-04-14

**Authors:** Jennifer Liao, Bita Asghari, Karen G. Carrasquillo

**Affiliations:** a New England College of Optometry, 424 Beacon St, Boston, MA, 02115, USA; b BostonSight, 464 Hillside Ave., Suite 205, Needham, MA, 02494, USA

**Keywords:** Stevens-Johnson syndrome, Toxic epidermal necrolysis, PROSE treatment, Corneal opacity, Corneal neovascularization, Scleral lenses

## Abstract

**Purpose:**

To report two cases demonstrating the regression of corneal neovascularization and clearing of corneal opacification in patients with Stevens Johnson Syndrome (SJS) and Toxic Epidermal Necrolysis (TEN) undergoing prosthetic replacement of the ocular surface ecosystem (PROSE) treatment.

**Observations:**

Four eyes of 2 patients were analyzed. Regression of neovascularization and clearing of corneal opacification was observed in both patients. All 4 eyes demonstrated improvement in visual acuity with treatment. With treatment, both patients ultimately discontinued all prescribed topical therapies. It was discovered upon review of these cases that all 4 eyes were managed with PROSE devices designed with back-surface channeled haptics.

**Conclusions and Importance:**

There currently is no known literature reporting on long-term regression of corneal neovascularization or clearing of corneal opacity in SJS or TEN patients with the use of scleral prosthetic devices. This report of 2 cases highlights the improvement in corneal function with PROSE treatment involving the use of channeled designs in patients with SJS or TEN. More research is needed to better understand how PROSE or scleral lens design features affect patient outcomes and why some patients may show regression in corneal neovascularization.

## 1 Introduction

Stevens-Johnson syndrome (SJS) and its more severe form, Toxic Epidermal Necrolysis (TEN), are parts of the same spectrum of a severe immunologic condition characterized by blistering of the skin and mucous membranes.^1^^,^^2^ SJS is a limited form, characterized by mucous membrane erosions and blisters on a limited area of the skin while TEN involves a confluence of blisters and erosions in more than 30% of the total body surface area.^3^ SJS/TEN cause severe inflammation that can target multiple organ systems, including the ocular, oral, respiratory, gynecological, gastrointestinal and integument systems.^1^, ^4^ Although considered a rare disease, with an estimated range of 0.04–7 cases per million population, the effects of SJS and TEN are widespread in affected patients and associated with high mortality and long-term morbidity.^1^^,^^5^, ^6^, ^7^

A significant majority of patients with SJS/TEN will have ocular pathology.^2^^,^^8^, ^9^, ^10^, ^11^, ^12^ Retrospective studies by Power et al. (1995) and Chang et al. (2007) report ocular involvement to be 69% and 81% respectively in patients with SJS, and 50% and 67% respectively in patients with TEN.^13^ Ocular pathology is believed to be caused by apoptosis and necrosis of epidermal layers followed by intense inflammation, most commonly affecting ocular surface tissues, bulbar and palpebral conjunctiva, lid margins, eyelashes and eyelid skin.^2^ Destruction of these ocular surface tissues subsequently results in ocular surface disease, ranging from mild cases with conjunctivitis to severe cases of destructive inflammation causing mucous membrane sloughing, symblepharon formation, and later-stage ocular surface cicatrization. Dry eye syndrome is the most reported late ocular complication of SJS/TEN, affecting 42–59% of cases, with accompanying symptoms of varying pain, photophobia and, in severe cases, permanent vision loss, which can greatly negatively impact a patient's function and quality of life.^1^^,^^12^^,^^14^ Ocular complications have been acknowledged as the most debilitating chronic effect of SJS/TEN.^1^

Management of patients living with chronic ocular complications from SJS/TEN has historically included prophylactic and palliative management. These approaches include infection prevention, management of pain and photophobia, and controlling inflammation.^15^ These interventions can be challenging, and the results are variable.^3^ Medical management of ophthalmic manifestations may include the use of topical lubrication, topical steroids, autologous serum, cyclosporine or lifitegrast. Surgical or mechanical management include punctal occlusion, amniotic membrane transplantation, fornix reconstruction, mucous membrane grafting, and eyelid procedures.^2^, ^3^, ^5^^,^^16^ Although many patients with chronic ocular manifestations of SJS/TEN can be successfully managed with palliative, medical or surgical intervention, a subset of patients continue to suffer from vision loss and varying amounts of ocular discomfort despite these interventions.

Management of ocular complications from SJS/TEN with prosthetic replacement of the ocular surface ecosystem (PROSE) has been described in various cases in the literature to be a safe and effective treatment for vision rehabilitation and visual function improvement, especially when medical or surgical management is unsuccessful.^14^, ^16^, ^17^, ^18^, ^19^, ^20^^,^^21^ PROSE treatment involves using a custom fabricated, large-diameter, gas permeable lens (previously known as the Boston Scleral Prosthetic Device^22^) to vault the cornea and cover the underlying ocular surface in preservative-free saline. As a result, PROSE treatment provides constant lubrication to the corneal surface, provides a smooth refractive surface, and protects the corneal surface from mechanical lid-forces with blinking, which allows for therapeutic and visual benefits.^14^, ^15^^,^^17^^,^^23^^,^^22^ In addition to the well-established therapeutics benefits, reports stemming from our center and others in the literature have found an association between SJS and ectasia.^24^, ^25^, ^26^ This finding also sheds light into the requirements for proper visual rehabilitation in these patients. These corneas often manifest higher order aberrations (HOA), requiring front surface aspheric curves or custom HOA correction to mitigate such aberrations. PROSE devices can be customized with varying degrees of front surface eccentricity which provide aberration control, which can be helpful for this patient population.^21^^,^^27^

This case series discusses two patients with SJS or TEN who were managed with continuous daily PROSE wear and demonstrated improvement in ocular physiological function, as noted by the clearing of corneal opacification and regression of corneal neovascularization. All four eyes in these two cases happened to be treated with PROSE device designs that include a novel back-surface channel technology, a design feature intended to increase tears exchanged between the post-lens reservoir and external lens environment, as well as decrease suction of the lens on eye. Improvement in corneal opacification and neovascularization was observed by Sotozono et al. (2014) in patients with SJS/TEN after wearing smaller limbal rigid contact lenses of 13.0–14.0mm for 3 months.^28^ To the best of our knowledge, there are no publications reporting on the long-term improvement in corneal neovascularization, opacification or scarring, nor have there been reports of improvement in long-term ocular surface function in SJS/TEN affected eyes treated with PROSE or scleral lenses. This case series would be the first to report these clinical findings.

## 2 Methods

This was a retrospective case series deemed exempt from IRB review by New England Institutional Review Board, as under BFS-KC-Retrospective-01. In each case, informed consent regarding risks and benefits of treatment was obtained from the patient or legal guardian. Accordingly, all guidelines were followed to ensure HIPAA compliance. We adhered to the Declaration of Helsinki and applicable federal and state laws.

We undertook a retrospective analysis of medical records from the PROSE treatment clinic at BostonSight in Needham, Massachusetts from July 2018 to October 2019. The inclusion criteria were improvement of a corneal opacity and regression of corneal neovascularization in patients managed with SJS/TEN. The cases fitting this inclusion criteria involved the use of novel channels on the back surface of PROSE device haptics.

**Fig. 1:**
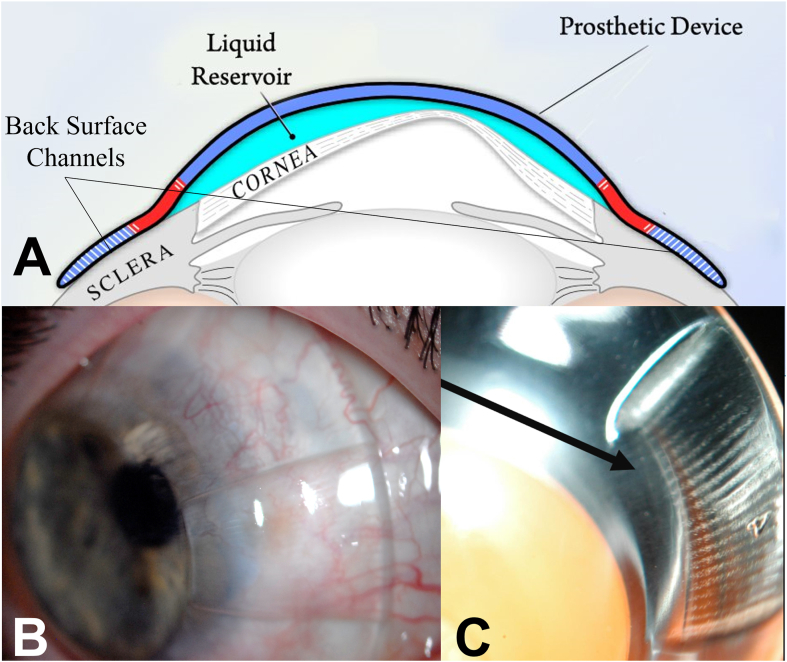
(A) A schematic of a PROSE device with back-surface channels in the haptics of the device on eye. (B) A PROSE device with a prominent channel viewed under diffuse white light slit lamp under low magnification. (C) A high magnification, diffuse white light slit lamp image of a PROSE device on a plunger with an arrow pointing to a channel, seen as back-surface grooves.

PROSE treatment involves the design and custom fabrication of FDA-approved prosthetic devices for therapeutic use on a daily wear basis as reported previously.^14^, ^16^, ^17^, ^18^, ^19^ Assessment of physiological function included evaluation of corneal clearance, haptic alignment, fluid ventilation, and corneal response through slit lamp examination, as well as subjective tolerance after various hours of wear. Subjective tolerance was assessed after 1, 3–4, and 6–8 hours of PROSE device wear. Customizations of PROSE devices, including addition of back-surface channels, were added as needed to optimize physiologic function by increasing fluid ventilation and/or reducing suction with wear (Fig. 1A–C). Routine photo-documentation of corneal findings using an RS-1000 Zoom Slit Lamp digital photo unit with a mounted Nikon D200 camera was an integral part of clinical assessment. The Efron grading scale was used for evaluation of corneal staining, while grading of neovascularization and opacification was assessed using the grading system proposed by Sotozono et al. (2007) for the evaluation of chronic ocular manifestations in patients with Stevens–Johnson Syndrome.^29^ Patients returned for evaluation of medical status and monitoring of device function at various follow-ups and intervals, ranging from 1 to 17 months, as appropriate for each case.

### 2.1 Case report 1

A 19-year-old Caucasian female with a history of SJS due to a reaction to oral sulfamethoxazole/trimethoprim one year prior, was referred to the BostonSight clinic for consultation. The patient presented with complaints of pain and fluctuating vision in both eyes, with the left eye worse than right. Ocular history was remarkable for recurrent infectious corneal ulcers, occurring more frequently in her left eye, and a history of ocular management with artificial tears, ophthalmic antibiotics, bandage soft contact lenses and cryo-preserved amniotic membrane. The patient also had a history of lash electrolysis on both eyes for trichiasis. At the time of her first evaluation, the patient was using artificial tears four times a day and liquid gel drops at bedtime for both eyes.

**Fig. 2:**
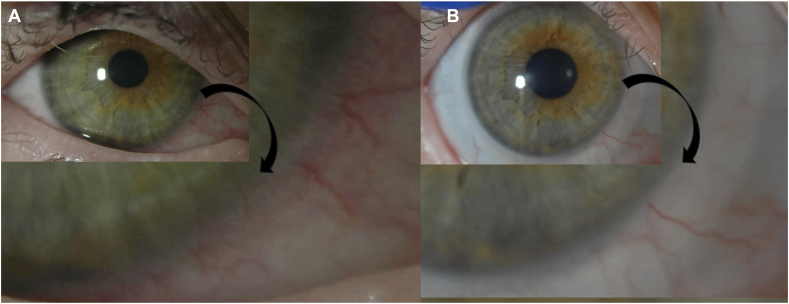
Diffuse white light images in low and high magnification of the right eye of patient described in Case 1 at baseline (A), with mild peripheral corneal neovascularization. Regression and resolution of peripheral corneal neovascularization is visualized at 8 months (B) following continued daily wear of a fluid-ventilating PROSE design with back-surface channels.

**Fig. 3:**
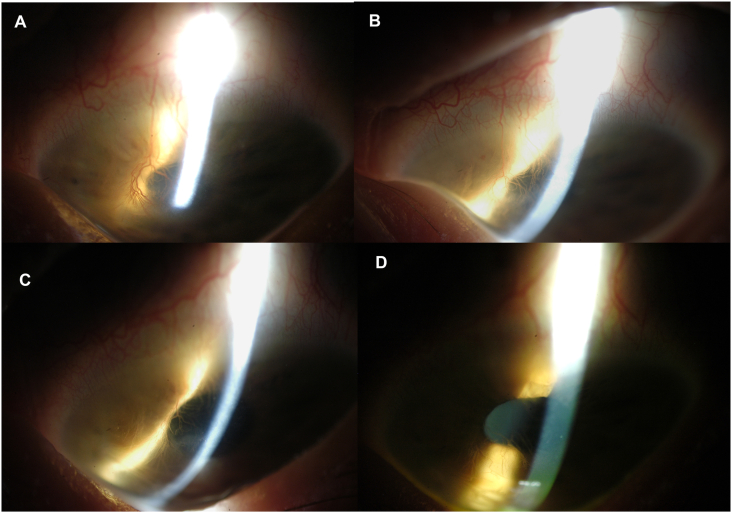
(A) Corneal neovascularization and haze of the left eye observed by indirect illumination at baseline of patient in case 1. (B) Progressive regression of corneal neovascularization and haze is visualized at 1 month (C), 4 months (D), and 8-month (D) follow-up, with daily wear of a PROSE device designed with back surface channels.

Entering visual acuity was 20/20 in her right eye, and 20/40^+1^ in her left eye, with pinhole potential to 20/30^−1^. Baseline slit lamp examination revealed meibomian gland atrophy with keratinized and inflamed lid margins and scarring of the upper palpebral conjunctiva on lid eversion in both eyes. All four puncta were patent. The right cornea had a small temporal paracentral area of haze. There were also two round peripheral corneal opacities, and dense grade 2 punctate staining. The left cornea had grade 3 punctate staining. Corneal neovascularization for the right cornea revealed superficial (grade 0) neovascularization, documented as <1mm, inferior nasal (Fig. 2A) and the left eye cornea revealed dense grade 2 neovascularization into the visual axis (Fig. 3A), with scarring. Corneal opacification for the right eye was grade 0, clear cornea with iris details visible. The corneal opacification grading for the left eye was grade 1, with partial obscuration of iris details. The remainder of the eye examination was unremarkable for both eyes.

PROSE treatment was initiated in both eyes with goals of improving comfort, supporting the ocular surface, and improving vision. The patient was fit with a custom-fabricated device for each eye, which yielded best corrected visual acuity of 20/20^−1^ in the right eye and 20/20 in the left eye. Each device was designed with back-surface channels to facilitate tear exchange and lessen the chance of suction with device wear. The patient was advised to continue with daily device wear for both eyes and to return for follow-up.

The patient returned for follow-up at 1 month, 4 months, and 8 months following the start of her treatment. The right eye had regression of corneal neovascularization inferiorly and considerably less conjunctival injection (Fig. 2B). At each visit, there was notably more regression in corneal neovascularization (improving to grade 0) and haze (Fig. 3B–D) for the left eye. At 8 months, the cornea remained clear for the right eye, grade 0. The corneal opacification of the left eye improved from grade 1 to 0, with clear and visible iris details (Fig. 3D). Resolution of corneal punctate staining was also noted for both eyes. The patient averaged wearing her devices for 12–14 hours daily in both eyes comfortably. Best corrected visual acuity was 20/20^+2^ in the right eye and 20/15^−3^ in the left eye at the 8-month follow-up visit.

Over the course of the 8-month period that this patient was monitored, no topical steroids or prescribed ophthalmic medications were used, nor was there any surgical intervention. This patient continued with the use of preservative-free artificial tears as needed, and preservative-free lubricating gel at night for both eyes.

### 2.2 Case report 2

A 26-year-old African American male with a history of TEN due to a reaction to oral phenytoin five years prior, was referred to the BostonSight clinic. The patient presented with complaints of severe dryness, pain, foreign body sensation, burning, redness, and poor vision in both eyes. Ocular management at consultation included lubricating gel drops as needed and prednisolone acetate 1% four times daily for both eyes. The goals for PROSE treatment were to improve comfort, vision, and to support the ocular surface.

**Fig. 4:**
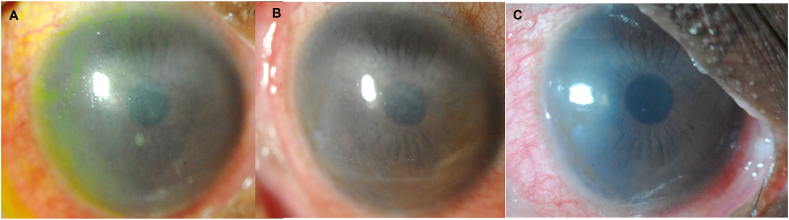
(A) Diffuse white light of the right cornea of patient in case 2 showing regression of central corneal haze at 5 months, (B) 10 months, and (C) 17 months of daily wear of a fluid-ventilating PROSE design with back-surface channels.

**Fig. 5:**
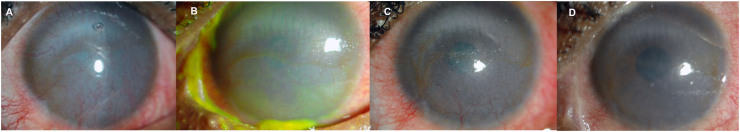
(A) Diffuse white light of the left eye cornea with diffuse corneal neovascularization and haze at baseline for case 2. Progressive regression in corneal haze and neovascularization is visualized at (B) 5 months, (C) 10 months, and (D) 17 months with daily wear of a fluid-ventilating PROSE design with back-surface channels.

Entering visual acuity with spectacles was 20/125^−1^ with pinhole potential to 20/50^+1^ for the right eye, and 20/250 for the left eye with pinhole potential to 20/100^−1^. Slit lamp evaluation revealed severe keratinization of the lid margins, bulbar conjunctiva, and palpebral conjunctiva for both eyes. A symblepharon was noted nasally in both the right and left eye, left eye worse than right. All four puncta were scarred. The right cornea revealed grade 2 neovascularization, while the left cornea was more severe with grade 3 neovascularization. Corneal opacification for the right eye was grade 2, with iris details poorly seen with pupil margin just visible (Fig. 4A). The corneal opacification for the left eye was grade 3, with complete obscuration of iris and pupil details (Fig. 5A). Corneal staining was diffuse and grade 4 for each eye.

The patient reported immediate improvement in comfort and vision with device wear in both eyes, with best corrected visual acuity improving to 20/30^−2^ in the right eye and 20/70 in the left eye. The devices were optimized for both eyes and dispensed for daily wear. The patient was advised to return to clinic for follow-up.

At the one-month follow-up, entering best corrected visual acuity was 20/60^+1^ for the right eye with pinhole to 20/50^+1^, and 20/80 for the left eye without improvement with pinhole. The patient complained of redness, discomfort, and transient blurred vision in both eyes during device wear. On the recommendation of his cornea specialist, the patient discontinued the use of prednisolone acetate drops one month after initiating PROSE treatment. He continued with the use of preservative-free artificial tears as needed.

Clinical evaluation revealed that both devices were moderately deposited with debris on the device surface as well as in the fluid reservoir, which was consistent with the patient's complaints of fluctuating vision. Corneal neovascularization and haze seen in both eyes at this visit were consistent with baseline findings. In efforts to improve the patient's comfort, back-surface channels were incorporated into the device haptic design to minimize suction of the device on eye and maximize tear exchange to the corneal surface.

The patient was monitored over a course of 17 months of daily device wear. Device wear was tolerated for an average of 12–14 hours per day for both eyes. Best corrected visual acuity with device wear fluctuated depending on the degree of debris on the device front surface or in the fluid reservoir during wear. At 17 months, best corrected visual acuity was 20/30^−2^ without pinhole improvement for the right eye and 20/80^+1^ with pinhole to 20/60 for the left eye.

Over the course of 17 months, the corneal surface showed signs of improvement in overall physiologic function, with significant regression in corneal neovascularization improving from grade 2 to grade 0 in the right eye and from grade 3 to grade 0 in the left eye. Corneal opacification for each eye improved to grade 1, with partial obstruction of iris details (Fig. 4, Fig. 5D). Corneal staining also improved to grade 2 in each eye.

Lifitegrast 5% was used nightly for both eyes for approximately two months early during PROSE treatment but was stopped due to poor tolerance and lack of effect. Prednisolone acetate drops had been discontinued one month after starting PROSE treatment. No other topical steroids or prescribed ophthalmic medications were used, nor was there need for surgical intervention. The patient continued with use of preservative-free artificial tears as needed over the course of treatment.

## 3 Discussion

Destruction of ocular surface tissues in SJS and TEN results in varying amounts of chronic debilitating discomfort, pain and vision loss in patients. The chronicity and consequential sequelae of damaged ocular surface tissues often demand long-term, rigorous management.^1^, ^2^, ^3^, ^5^^,^^19^ The goals of ocular management in chronic SJS/TEN have historically been infection prophylaxis, management of pain and photophobia and controlling ocular surface inflammation. These have commonly involved regular use of medical treatments, including lubricants, autologous serum, and topical anti-inflammatory medications. In more severe cases, especially when ocular sequelae worsen despite medical management, surgical intervention is necessary.

Over the past decade, PROSE treatment has been reported as safe and effective in providing palliative and visual rehabilitative care for severe ocular surface disease in SJS and TEN.^14^^,^^16^, ^17^ Multiple retrospective investigations and case series have reported increased visual outcomes, visual function, and quality of life in patients with SJS/TEN with PROSE treatment.^16^, ^18^, ^19^, ^20^, ^30^ A study by Tougeron-Brousseau et al. (2009)^15^ describes these therapeutic benefits in management with gas permeable scleral lenses. Sotozano et al. (2014) also demonstrates the therapeutic benefits with limbal rigid lenses.^29^

This case series describes two cases where improvement in ocular surface function was observed through the regression of corneal neovascularization and clearing of corneal opacities over the course of 8 months in a patient with SJS, and over the course of 17 months in a patient with TEN, when managed with PROSE treatment. Furthermore, topical medical management for ocular surface disease was no longer required for the second patient following PROSE treatment.

One of the most common causes of corneal neovascularization are inflammation of the eyelid and trauma,^31^ which are commonly occurring in SJS. As postulated previously, PROSE treatment provides an environment that supports healing and maintains integrity of the ocular surface. Once ocular surface integrity is established, inflammation is reduced – potentially paving the way for improvement in ocular surface functions.^32^ Shornack (2011) suggests that treatment with scleral lenses might reduce the risk of damage to surviving corneal epithelial cells.^33^ The protection and rehabilitation of surviving corneal limbal stem cells may potentially also contribute to contribute to decreased corneal neovascularization and opacity with scleral lenses.^28^

Channels, or non-penetrating grooves on the back surface of a lens, were historically used in scleral lens designs when low Dk and polymethyl methacrylate (PMMA) materials were limited in providing sufficient oxygen delivery to the ocular surface during lens wear.^34^^,^^35^ With the availability of high and hyper-DK materials today, as well as the capacity to adjust and customize haptics to fit scleral shape, the need for fenestrations and channels has decreased.^35^

Both cases in this series happen to have devices designed with back-surface channeled haptic designs. With ocular surface cicatrization, keratinization, corneal scarring and corneal limbal stem cell deficiency seen in chronic SJS/TEN, it is crucial to fit a PROSE device or scleral lens on the eye without stressing its existing compromised ocular surface tissue function. The addition of back-surface channels to PROSE device designs in these two cases was intended to optimize fit and comfort by increasing tear exchange and by decreasing suction on the eye with increased wear time. Literature has suggested that back-surface channels increase tear exchange, thereby contributing to increased oxygen exchange on the ocular surface.^23^, ^35^ Our assessment leads us to believe that channels not only increase oxygen exchange, as seen when fluorescein dyed tears flow from outside the device into the post-lens reservoir, but channels may also reduce physical and suctional forces of the lens on the eye, like breaking the seal of a suction cup. We theorize that the use of back-surface channels in a large diameter scleral lens in these two cases of SJS and TEN improved patient tolerance. Their role in the improvement in physiological function noted at the ocular surface in this case series is to be determined and needs to be further studied. It might be possible that the added allowance of oxygenated tears to the lens reservoir and the reduction of suction forces may contribute to further improvements in the ocular surface. Case series, such as this, may be subject to potential sources of bias. Our review of two SJS/TEN cases reflects a selection bias of patients who were deemed successful candidates for PROSE treatment based on improvement in comfort and vision on consultation and would not include cases determined to be poor PROSE candidates or those who did not wish to pursue PROSE treatment.

Severity of disease is also a variable to consider in our two cases. The ocular SJS severity for each case was assessed with the grading scale from Sotozono et al. (2007),^29^ which grades severity of 13 ocular surface components of SJS manifestations, scored in severity from 0 to 3, 3 being the most severe. The 13 components include: corneal punctate staining, corneal epithelial defect, loss of palisades of vogt, conjunctivalization, neovascularization, opacification, corneal keratinization, hyperemia, symblepharon formation, trichiasis, mucocutaneous junction involvement, meibomian gland involvement, and punctal involvement. The total score is the sum of all 13 components, ranging from 0 to 39. Based on this grading system, case 1 had a total score of 11/39 in the more severe eye and case 2 had a total score of 24/39 based on the more severe eye at baseline. While Sotozono et al. does not define disease severity based on the total score, the patient in case 2 can be described as more severe than the patient in case 1, and we can hypothesize and infer they would be considered mild (case 1) and moderate (case 2) in the spectrum of disease severity.

The mild and moderate severity of our cases limits our possibility to conclude or hypothesize if PROSE treatment may provide the same improvement in more severe cases. It also may suggest that regression of corneal opacification and neovascularization in PROSE treatment may be more likely in mild or moderate cases of SJS/TEN.

As mentioned above, the effect, if any, of back surface channels needs to be further studied. Our observation that both cases involved treatment with back-surface channel designed PROSE devices may be a consequence to selection bias as well, given the specificity of case selection and approaches of the PROSE fitter. Lastly, although literature searches revealed no reports of long-term (more than 6 months) improvement of clinical signs in SJS/TEN patients with scleral lens use, there is the possibility that photo documentation was not available in other retrospective studies to assess these changes over time.

Despite its limitations, this case series highlights two novel findings in the management of ocular pathology in SJS/TEN: the long-term improvement in clinical signs of corneal opacification and neovascularization with PROSE treatment, and observation of these improved clinical signs during treatment with back-surface channel designed PROSE devices.

With multiple literature sources supporting the benefits and efficacy of PROSE and scleral lens treatments in SJS/TENs, as well as with technological advances to PROSE and scleral lens customizability, understanding ways to improve ocular surface function and clinical signs in SJS/TENs management may be the first step to optimizing long-term ocular management in patients suffering from this chronic, debilitating disease. Although fluid-ventilation with channels and air ventilation with fenestrations have been increasingly viewed as unnecessary in the age of hyper Dk materials with higher oxygen permeability, the use of channels in this case series highlights that they can continue to be positively impactful on patient outcomes. More research is needed to specifically examine how PROSE or scleral lens management can improve ocular surface function, and whether back-surface channels play a key role in these outcomes.

## Patient consent

Consent to publish this case report has been obtained from the patient(s) in writing. This report does not contain any personal identifying information.

## Disclosures

No financial disclosures

Neither author has a proprietary interest in PROSE treatment.

## Funding

No funding received for this work.
